# Evaluation of a Novel Spine and Surface Topography System for Dynamic Spinal Curvature Analysis during Gait

**DOI:** 10.1371/journal.pone.0070581

**Published:** 2013-07-23

**Authors:** Marcel Betsch, Michael Wild, Brian Johnstone, Pascal Jungbluth, Mohssen Hakimi, Britta Kühlmann, Walter Rapp

**Affiliations:** 1 Oregon Health & Science University, Department of Orthopaedics and Rehabilitation, Portland, Oregon, United States of America; 2 Klinikum Darmstadt, Department of Trauma and Orthopaedic Surgery, Darmstadt, Germany; 3 University Hospital Duesseldorf, Department of Trauma and Hand Surgery, Duesseldorf, Germany; 4 University Hospital Tuebingen, Department of Sports Medicine, Tuebingen, Germany; University of Ulster, United Kingdom

## Abstract

**Introduction:**

The assessment of spinal deformities with rasterstereography can enhance the understanding, as well as can reduce the number of x-rays needed. However, to date this technique only allows measurements under static conditions. Since it would be of great value to be able to also analyze the spine in dynamic conditions, the present study evaluated a novel rasterstereographic system.

**Materials and Methods:**

A new rasterstereographic device was evaluated in a comparison with the gold standard in motion analysis, the VICON system. After initial testing using 12 flat infrared markers adhered to a solid plate, the two systems were evaluated with the markers adhered onto the backs of 8 test subjects. Four triangles were defined using the markers, and the sides of each triangle were measured under static and dynamic conditions.

**Results:**

On the solid plate, the sides of the 4 triangles were measured with a measuring tape and then by the two optical systems. Rasterstereography showed a high accuracy in marker detection on the solid plate. Under dynamic conditions, with the subjects walking on a treadmill, the rasterstereographically-measured side lengths were compared with the lengths measured by the VICON system as an assessment of marker detection. No significant differences (p>0.05) were found between the systems, differing only 0.07–1.1% for all sides of the four triangles with both systems.

**Discussion:**

A novel rasterstereographic measurement device that allows surface and spine topography under dynamic conditions was assessed. The accuracy of this system was with one millimeter on a solid plate and during dynamic measurements, to the gold standard for motion detection. The advantage of rasterstereography is that it can be used to determine a three-dimensional surface map and also allows the analysis of the underlying spine.

## Introduction

The radiation burden of repeated whole spine x-rays, particularly in young patients with scoliosis, can lead to an increase in the breast and thyroid cancer risk, as well as to an increase in the leukemia rate in this population [1], [2], [3], [4]. In addition to the two-dimensional information taken from x-rays, a three-dimensional understanding of spinal deformities is seen as important in the treatment of these complex pathologies. For many years, optical surface measurement systems have helped surgeons to better understand the complexity of these deformities, and reduce the number of x-rays needed. The oldest optical technique to analyze the spine is called the Moiré topography, which uses interference patterns generated by a light source and a line grid on the back of the patient [5], [6]. Although, the sensitivity of this technique is good (74%), its false-negative values between 17–25% are not acceptable [7], [8]. Further research led to the invention of ultrasound or optical surface scanners, such as the Zebris, Quantec or Inspeck system, which allow a radiation-free and three-dimensional analysis of the back surface [9], [10], [11]. Most of these systems use the position of markers, placed on anatomical landmarks on the back surface, to reconstruct the spinal posture with adequate accuracy. However, these systems often require a trained operator because of the rather complicated setup of the components and placement of multiple surface markers on the back. In addition these systems do not allow a reconstruction of the underlying spine, which limits the use of these devices in many cases to experimental or research purposes [7].

In the 1980s, Hierholzer and Drerup developed a spine and surface topography system called rasterstereography [12], [13]. Rasterstereography is a method for stereophotogrammetric surface measurement of the back based on Moiré topography [14]. Horizontal parallel light lines are projected onto the unclothed surface of the back by a slide projector. A surface reconstruction of the back is then performed by transforming the lines and their corresponding curvature into a three-dimensional scatter plot. A 3D-model of the spine can then be calculated based on the specific convex shape of the spinous process of the vertebra prominence (VP) and the concavity of the lumbar dimples as fixed points. Transverse and sagittal profiles, the spinous process line and several spinal angles and indices can be analyzed with rasterstereography. Furthermore, it is possible to use the two lumbar dimples to determine pelvic obliquity, because they are in close relation and fixed to the underlying posterior superior iliac spines [15], [16]. From the orientation vectors of the skin surface over the lumbar dimples, it is also feasible to draw conclusions about the pelvic torsion around the transverse-axis [13], [16]. In contrast to all other optical measurement devices, rasterstereography allows an analysis not only of the back surface, e.g. to assess cosmetic changes due to the deformity, but also of the underlying spine. This is possible by the use of a spine model, which was created by Turner-Smith, based on x-rays of patients with scoliosis [17], [18].

In a series of studies, rasterstereography has proven to have high reliability and accuracy when compared to x-rays [13], [19], [20], [21]. However, it would be of great value to be able to also three-dimensionally evaluate the spine under dynamic conditions to provide a better understanding, of the rigidity of scoliosis or phenomena like the influence of pelvic obliquity and leg length inequality on the spine. Surgical interventions such as spinal fusions, and their effects on the mobility of individual spinal segments could also be better evaluated if dynamic measurements were possible. Thus, the purpose of this present study was to evaluate the accuracy of this spine and surface topography device under dynamic conditions.

## Materials and Methods

### Ethics Statement

The study was approved by the Ethics Committee of the University Hospital Tuebingen, Germany. All volunteers provided written informed consent to participate and were given the option to quit participation at any time.

### Measuring system

In a static situation the rasterstereography technique uses the automatic detection of the anatomical landmarks, VP and the two lumbar dimples, to calculate a biomechanical spine model with an accuracy of up to ±1 mm [13], [19]. However, even under static conditions, the use of infrared reflecting markers can sometimes be necessary, when the anatomy of these landmarks is being altered by subcutaneous fatty tissue, muscles or hair. For this, the anatomical landmarks have to be palpated and a respective marker has to be placed, which is then used in the calculation of the biomechanical spine model. Under dynamic conditions the movements of the skin and soft tissue above the anatomical landmarks during motion disturb the accurate detection of spinal landmarks. However, the use of infrared reflecting markers should minimize this problem. For rasterstereography, in preliminary work we determined that three markers (VP, lumbar dimples) are considered sufficient enough to calculate the position of the spine (data not shown).

The newly developed Formetric 4D motion spine and surface topography system (Diers International GmbH, Schlangenbad, Germany) was used in this study. This device is equipped with a digital network camera that allows measurements with a maximum frequency of up to 50 frames per second. The camera uses a CMOS sensor with a resolution of 1280×1024 pixels. Infrared reflecting markers (10 mm in diameter) are illuminated by the system from an array of 8 LEDs. The position of the markers can then be detected using an algorithm that scans the image for all bright elliptical regions on the back surface. From the position of the markers a sub-pixel approximation of the center of the marker is used to determine the exact position. With the help of these algorithms a complete reconstruction of the back surface can be performed in approximately 100 ms, allowing a real-time display of the three-dimensional reconstructed back and spine during measurements. We chose to evaluate the accuracy of this novel device in comparison with a three camera VICON system, which is widely used and considered the gold standard for motion analysis [22].

### Measuring setup

**Figure 1:**
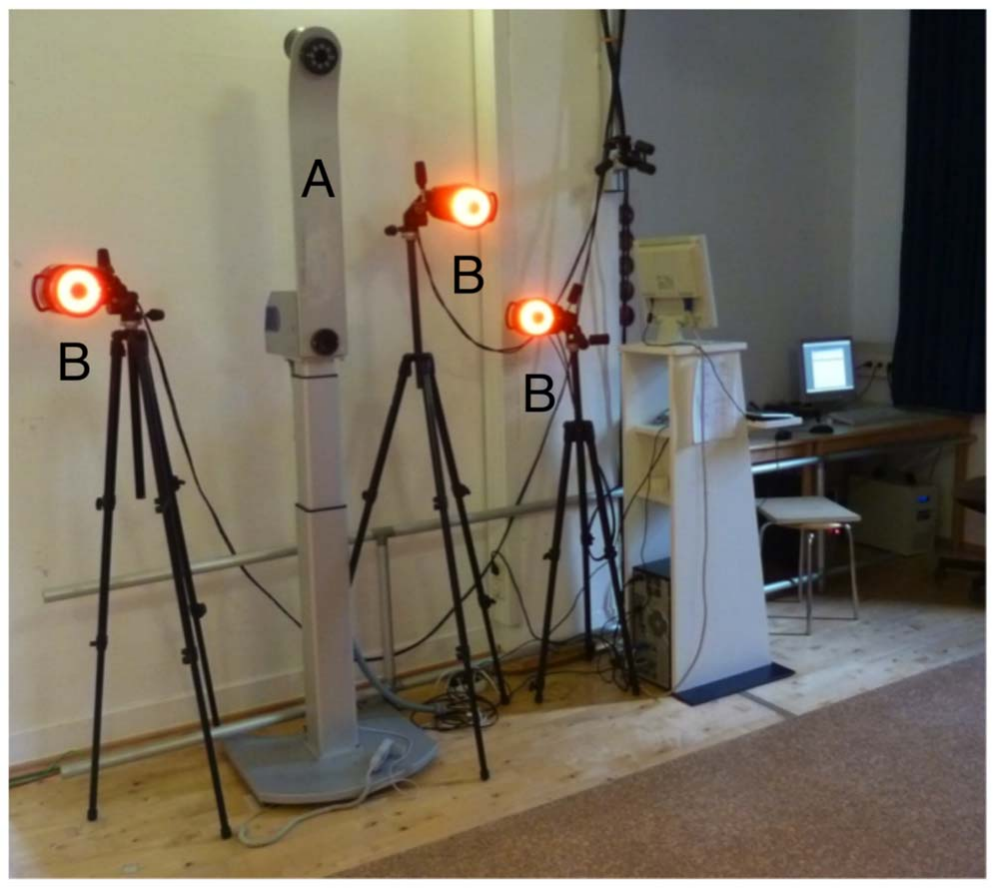
Setup of the measuring devices. The setup of the two measurement devices, placed 2 meters behind the measured test subjects. The rasterstereographic system is contained within a column (A), which contains the infrared-flash, the light projector and the digital camera. The VICON system consists of three cameras (B), each equipped with an infrared-flash for the marker detection.

The basic setup of both devices is shown in Figure 1. Both systems were placed 2 m behind the test subject since the rasterstereographic device is calibrated such that the projected light lines are focused at this distance. Three VICON cameras were placed at the same distance to the subjects (Figure 1). In pretests we determined that the infrared flash of the rasterstereographic device, which is used for the marker detection, does not interact with the flash of the VICON system. In general, automatic video analyzing systems like the VICON, use spherical markers in order to increase the projection angle of the visual field. However, these markers cannot be used in rasterstereography, because of the different technique used in the automatic markers detection. Therefore, flat infrared reflecting markers, with a diameter of 10 mm, were used.

**Figure 2:**
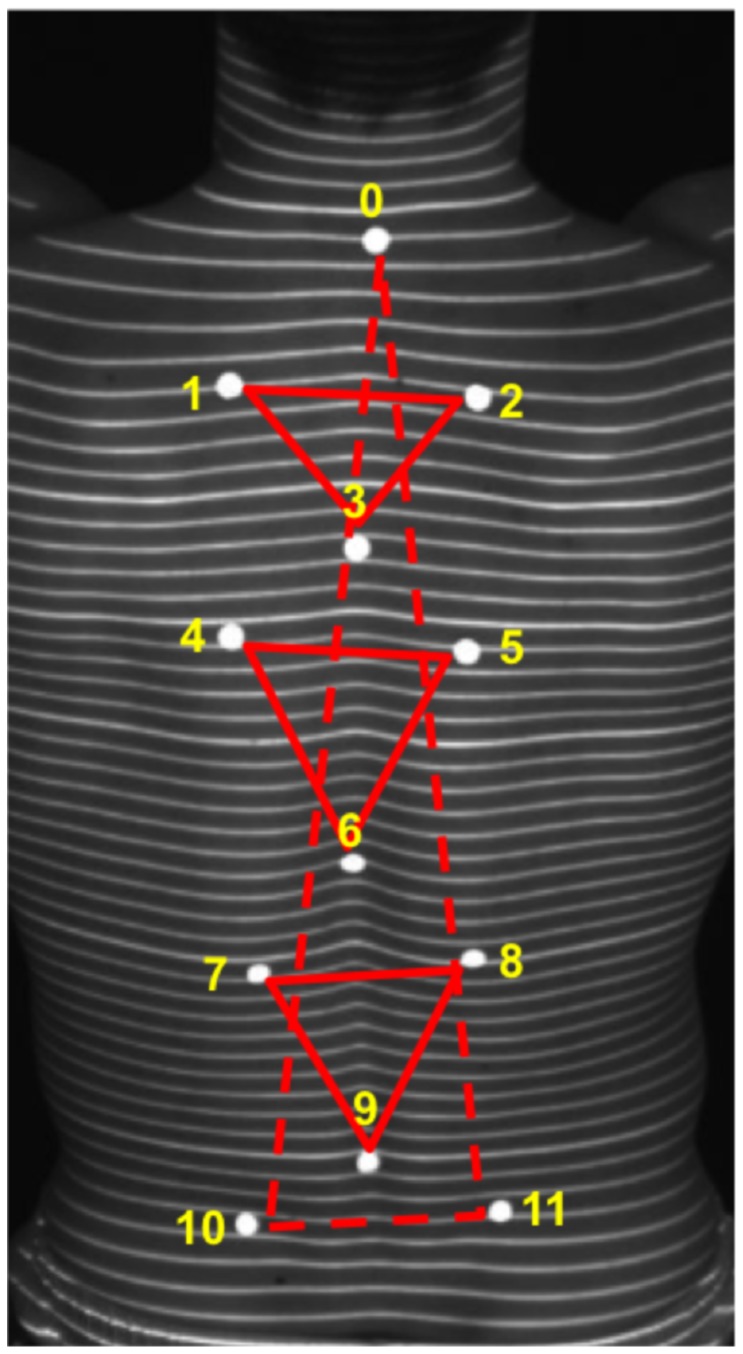
Marker placement on test subjects. The placement of the 12 markers (white dots) on the back of a measured test subject are shown. The markers were placed so that they formed four triangles, of which the sides were determined by the detection of the markers by the tested systems. Triangle 1 (T1): between the scapula, defined by M1, M2, M3. Triangle 2 (T2): below the scapula, the thoracic spine (M4, M5, M6). Triangle 3 (T3): the lumbar spine (M7, M8, M9). Triangle 4: from the VP to the two lumbar dimples (M0, M10, M11).

The accuracy of the marker detection in both systems was tested in two formats, firstly under static conditions using a flat plate and then secondly under static and dynamic conditions with 8 volunteers. A total of 12 flat infrared markers were used in both formats (Figure 2). The 12 markers were placed so that the connecting lines between the markers defined 4 triangles. The accuracy of the automatic marker detection was evaluated in a first test on a static flat plate to demonstrate the accuracy of both systems under static conditions. The sides of the 4 triangles were measured by hand with a measuring tape and by the automatic detection of the marker positions using the rasterstereographic and VICON system. After this testing the markers were placed on the spinous processes of the 7^th^ cervical vertebra (M0), 6^th^ thoracic (M3), 12^th^ thoracic (M6), 5^th^ lumbar vertebrae (M9) and on the two lumbar dimples (M10, M11) of eight test subjects (age: 24.63±1.3 years; height: 1.78±0.07 m; weight: 74.38±8.09 kg). In order to form the above-mentioned triangles, additional markers (M1, M2, M4, M5, M7 and M8) were placed laterally to the spinous process line at least 50 mm apart from each other. Based on the position of the markers, the following four triangles were defined. Triangle 1 (T1): between the scapulae, defined by M1, M2, M3. Triangle 2 (T2): below the scapula, on height of the thoracic spine (M4, M5, M6). Triangle 3 (T3): on height of the lumbar spine (M7, M8, M9). Triangle 4: from the VP to the two lumbar dimples (M0, M10, M11).

Each subject was measured once with arms hanging on the sides for six seconds with both systems while standing. To produce dynamic conditions the test subjects walked on a treadmill (HP Cosmos, Germany), which was placed in front of the measuring devices. All subjects adapted to the respective velocity of the treadmill for one minute before the simultaneous measurement for six seconds with both systems was started. The marker detection of the two systems was evaluated while the subjects were walking at three different speeds (1.5 km/h, 3 km/h, 6 km/h) on the treadmill.

### Data analysis

The coordinates of all 12 markers, from both measuring systems and in both formats, were used to calculate the lengths of the sides of each of the four triangles. Based on the calculated sides of each triangle the differences between the rasterstereographic and the VICON system were analyzed. The differences between the tape measurements and the two optical devices are expressed in millimeters and as the percentage of deviation from the tape-measured lengths (Table 1). A negative value means that the lengths measured by the VICON system were larger than those measured by the rasterstereographic device, and a positive value means that the lengths were longer when using the rasterstereographic device than the VICON system. For the dynamic analysis a tape measurement was not possible during motion. Therefore, the results of the rasterstereographic marker detection were compared directly with the gold standard the VICON system. All differences found between the two systems are expressed in millimeter and in percentage of deviation from the VICON measurements (Table 2).

**Table 1:** Distances on a solid plate.

		Distances			Absolute differences in mm and in percent		
Triangle	Markers	Tape measured	Formetric	Vicon	Tape-Formetic	Tape-Vicon	Formetric-Vicon
1	M1–M2	121	121.00	121.16	0.00 (0.00%)	−0.16 (0.13%)	0.17. (0.14%)
	M1–M3	137	135.57	137.26	1.43 (1.04%)	−0.26 (0.19%)	−1.69 (1.25%)
	M2–M3	137	135.23	137.73	1.77 (1.29%)	−0.73 (0.53%)	−2.51 (1.86%)
2	M4–M5	151	150.62	151.17	0.38 (0.25%)	−0.17 (0.11%)	−0.54 (0.36%)
	M4–M6	140	139.67	141.46	0.33 (0.24%)	−1.46 (1.04%)	−1.79 (1.28%)
	M5–M6	150	150.21	150.84	−0.21 (0.14%)	−0.84 (0.56%)	−0.63 (0.42%)
3	M7–M8	98	98.20	98.79	−0.20 (0.20%)	−0.79 (0.81%)	−0.59 (0.60%)
	M7–M9	70	70.23	70.78	−0.23 (0.33%)	−0.78 (1.11%)	−0.56 (0.80%)
	M8–M9	70	71.35	70.83	−1.35 (1.93%)	−0.83 (1.19%)	0.53 (0.74%)
4	M10–M11	149	149.01	148.66	−0.01 (0.01%)	0.34 (0.23%)	0.35 (0.23%)
	M0–M10	644	643.70	646.46	0.30 (0.05%)	−2.46 (0.38%)	−2.76 (0.43%)
	M0–M11	646	646.72	646.64	−0.72 (0.11%)	−0.64 (0.10%)	0.08 (0.01%)
				Mean	0.13 (0.01%)	−0.73 (0.49%)	−0.86 (0.51%)
				SD	0.84 (0.79%)	0.71 (0.46%)	1.09 (0.71%)

For each of the four triangles the distances between the markers were measured by hand using a measuring tape, by the VICON system and by the novel spine and surface topography system. The differences between the measurements are shown in millimeters as well in percentage of the deviation from the actual tape measured side lengths. A total of 12 side lengths of four triangles were measured resulting in a mean difference between the tape measurements and rasterstereography of 0.13±0.84 mm. The difference was −0.73±0.71 mm between tape and VICON measurements and −0.86±1.09 mm between rasterstereography and the VICON system.

**Table 2:** Side lengths measured with the two devices during motion.

		Differences												
Velocity	Variable	Triangle 1			Triangle 2			Triangle 3			Triangle 4			Mean
		Seg 1	Seg 2	Seg 3	Seg 1	Seg 2	Seg 3	Seg 1	Seg 2	Seg 3	Seg 1	Seg 2	Seg 3	
0 km/h	Mean	−.78	−.60	−1.41	−.58	−.08	−.02	−.43	−.52	−.89	−.87	3.61	−.26	−.24
	SD	1.04	.94	1.24	1.22	2.35	2.33	.74	1.21	1.25	1.83	3.10	.21	
	Max	1.67	.26	.42	.81	3.51	3.25	.41	1.12	.82	.02	7.77	.02	
	Min	−2.20	−2.19	−3.86	−3.58	−4.66	−5.30	−2.18	−2.94	−2.59	−5.71	−.08	−.62	
1.5 km/h	Mean	−1.26	−1.22	−1.47	−.45	.53	.25	−.25	.42	.42	−.49	3.65	−.49	−.03
	SD	.66	.58	.84	.68	.82	.55	.36	.74	.45	.23	3.92	.23	
	Max	−.45	−.41	−.65	.72	1.74	1.29	.29	1.39	1.30	−.33	7.11	−.33	
	Min	−2.52	−2.13	−3.46	−1.79	−.57	−.50	−.94	−.65	−.30	−1.05	−2.53	−1.05	
3.0 km/h	Mean	−.93	−1.10	−1.35	−.51	.72	.42	−.43	.32	.08	−.50	4.19	−.50	.03
	SD	.56	.62	.78	.49	.74	1.12	.57	.66	.77	.22	2.74	.22	
	Max	−.22	.07	−.32	.13	1.70	2.09	.58	1.24	1.06	−.18	7.05	−.18	
	Min	−2.20	−2.06	−2.71	−1.48	−.46	−.70	−1.47	−.95	−.87	−.80	−1.83	−.80	
6.0 km/h	Mean	−1.24	−1.28	−1.29	−.96	.45	.44	−.47	.07	.04	−.48	2.84	−.48	−.20
	SD	.38	.70	1.23	.53	.73	.70	.34	.70	.62	.21	3.77	.21	
	Max	−.76	−.27	.59	−.01	1.29	1.45	.06	1.07	1.07	−.10	5.87	−.10	
	Min	−2.03	−2.43	−3.20	−1.99	−.83	−.86	−.91	−1.14	−.84	−.76	−4.55	−.76	
Mean		−1.05	−1.05	−1.38	−.63	.40	.27	−.40	.07	−.09	−.59	3.57	−.43	
		0.92%	0.85%	1.1%	0.55%	0.32%	0.21%	0.39%	0.07%	0.08%	0.12%	0.77%	0.41%	

The differences between the marker detection of the rasterstereographic device and the VICON system are shown in absolute numbers (millimeter) as well as in percent deviation (%) for all four triangles, including all segments of the 4 triangles during standing (0 km/h) and walking speeds of 1.5, 3 and 6 km/h.

### Statistical analysis

All data are presented as mean values with standard deviations or 95% confidence levels. Paired T-tests were used to evaluate for any statistical differences in the measured values between the methods. The level of significance was set at p<0.05. Statistical analysis and graphical presentation were done using SPSS® (Version 20.0, IBM, USA) software.

## Results

On the flat plate under static conditions, the sides of the four triangles were measured by hand and by the two motion analysis systems. The results of the marker detection for each segment and all devices are shown in Table 1. The smallest differences were found between the tape measurements and the rasterstereographic system with a mean value of 0.13±0.84 mm (mean value ± standard deviation) (*p>0.993*) which is a 0.013% deviation from the actual sides of all four triangles. The mean difference between the tape measurements and the VICON measured distances was 0.73±0.71 mm (or 0.49% deviation of the actual side lengths), and this difference was also not significant (*p>0.993*). We measured a mean difference in the automatic marker detection for all triangles of −0.86±1.06 mm between the two devices, which is a 0.51% deviation of the rasterstereographic results from those with the VICON system. However, the measured difference between the two devices was also not significant (*p>0.05*).

Eight volunteers were measured under static and dynamic conditions with 12 flat infrared-reflecting markers on their backs. The mean differences for each triangle and for each condition, measured with the rasterstereographic or the VICON system, are shown in Table 2. The percentages of the deviation of the rasterstereographically-measured lengths from the VICON system measured lengths are included in Table 2.

**Figure 3:**
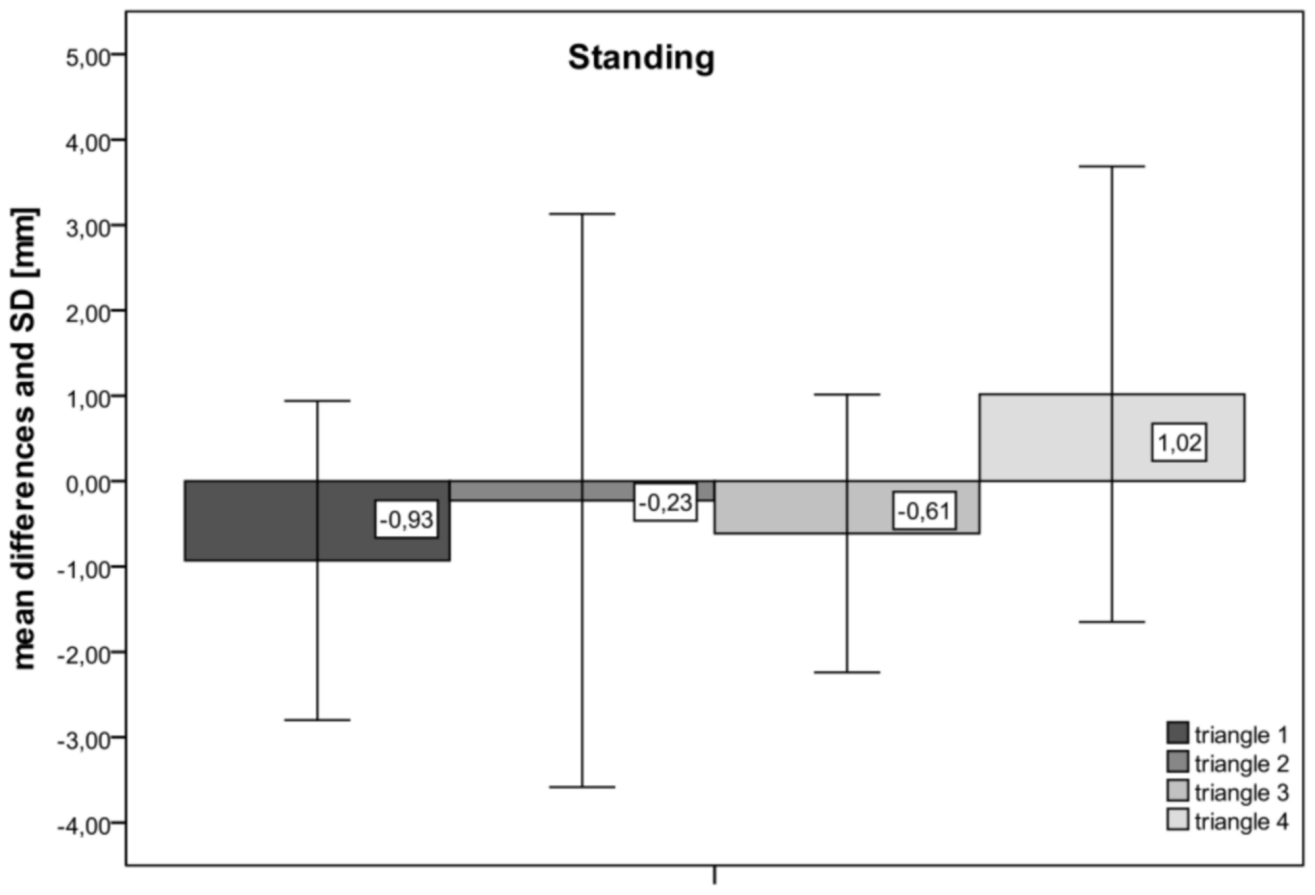
Accuracy of marker detection under static conditions. The mean differences of the measured sides of the four triangles during standing. The smallest differences in the marker detection were found in triangle 2 with a difference of −0.23 mm and the biggest differences were found in triangle 4 with a difference between the two systems of 1.02 mm. No measured side lengths of the triangles differed significantly.

**Figure 4:**
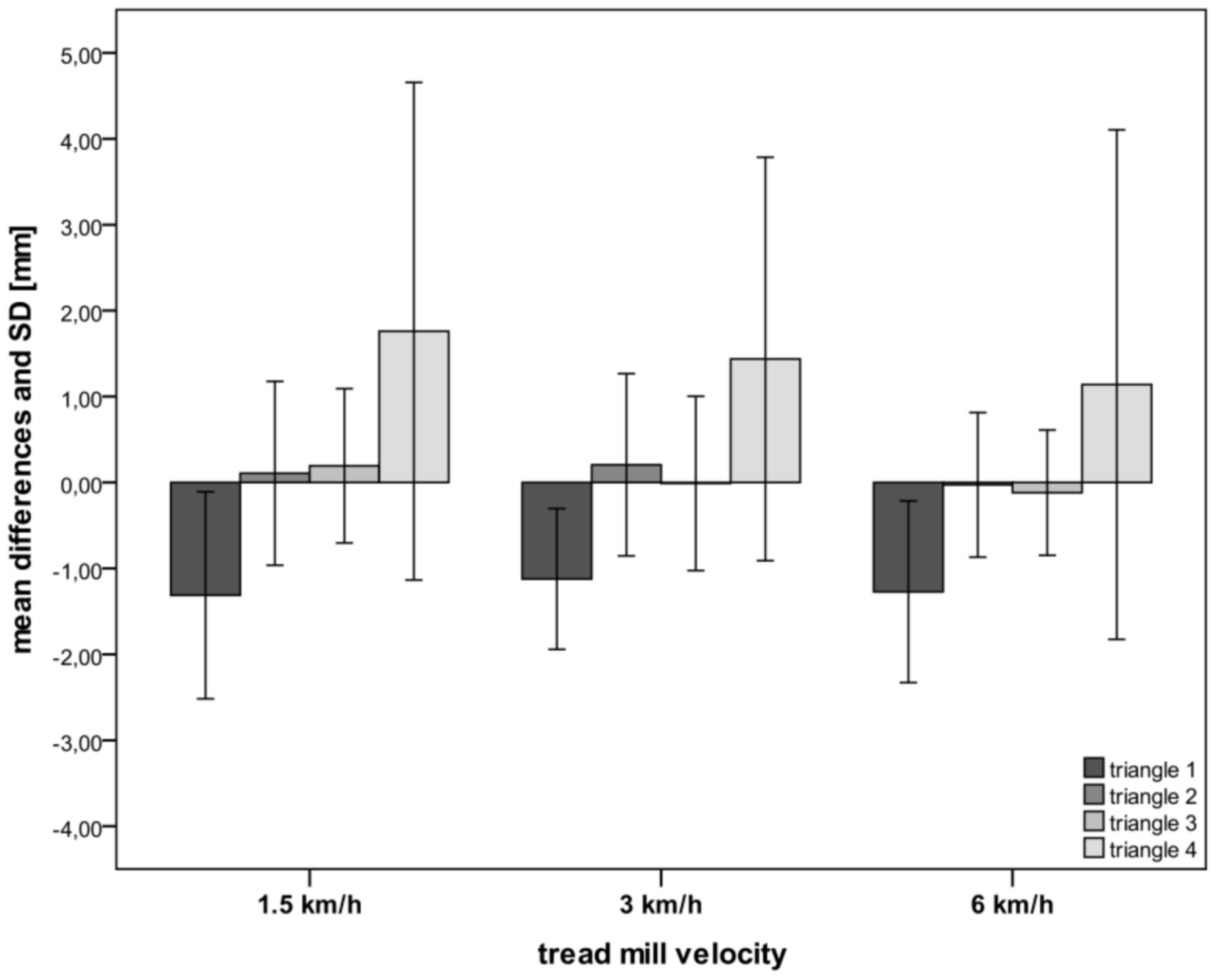
Accuracy of marker detection under dynamic conditions. The measured sides of all four triangles during motion with subjects walking at speeds of 1.5, 3 and 6 km/h. There were no significant differences (p>0.05) in the measured segments of the triangles between the two systems.

For triangle 1 we found mean differences (all speeds combined) for the three sides between 1.05–1.38 mm, which corresponds to a deviation in percentage between 0.85–1.1%. For triangles 2 and 3 the mean differences between the two optical devices ranged between 0.07 to 0.63 mm, which corresponds to a 0.07–0.55% deviation. Triangle 4 had the longest sides with lengths of up to 473 mm. The mean differences between the two systems ranged between 0.43–3.57 mm. When these results are expressed in percent of deviation from the total sides measured with the VICON system, the deviation was only between 0.12–0.77%. No statistical difference (p>0.05) was found for all dynamic measurements between the rasterstereographic and the VICON system. The mean difference between the two systems for all four triangles during standing was −0.24±1.46 mm. In dynamic conditions, the mean difference at 1.5 km/h was −0.03±0.84 mm, for 3 km/h it was 0.03±0.79 mm and for 6 km/h it was −0.20±0.844 mm, indicating that the speed of the treadmill did not influence the accuracy of the marker detection. The accuracy of the marker detection, in all four triangles during standing and in dynamic conditions is also shown in Figure 3 and Figure 4.

## Discussion

Rasterstereography has evolved from a static measuring technique into a system that is able to analyze the spine and back surface under dynamic conditions. The purpose of this study was to evaluate the accuracy of marker detection with this new dynamic rasterstereographic system. Since the VICON technique is considered the gold standard in motion analysis, we compared the marker detection of rasterstereography with a three camera VICON system. When comparing the results of the static measurements, it must be noted that the distance differences between the markers should not be analyzed at a level beyond the precision of the reference method (tape measurements ±1 millimeter). Therefore, we conclude that the results of our study show that the marker detection of the rasterstereographic technique under static conditions is within one millimeter of the tape-measured lengths and that the accuracy between rasterstereography and the VICON method did not differ significantly under static conditions. In the second format of our study we measured the sides of four triangles on the backs of eight volunteers. Under static and dynamic conditions the lengths of the sides of all four triangles could also be accurately measured within one millimeter (1.5%) of the VICON system.

Since the projected light lines, which are used to analyze the back surface, are focused at a distance of 2 m with the rasterstereographic device, patients have to walk on a treadmill during analysis to keep that distance. However, when measuring with the VICON system, the range and number of the cameras also limits the analysis. Reflective markers are commonly used for motion analysis. The positioning of the markers on the skin above anatomical landmarks is considered one of the major problems of motion analysis influencing the accuracy of systems that use markers. In recent studies using fluoroscopy or MRI, errors of marker measurement, due to skin to bone movements on e.g. the thigh are in a range of ±31 mm for flexion/extension and up to 15° for rotation [23], [24]. Skin to bone artifacts have to be considered for all marker based motion analysis systems and methods to correct these soft tissue artifacts have to be developed [23]. That stated, since fluoroscopy, CT and MRI cannot be used routinely in motion analysis because of the radiation and the cost of these devices, there is a place for optical surface analysis systems in the clinical arena. We believe that data on the kinematic function of the spine could be of great clinical value, especially in the diagnosis and treatment of spinal pathologies such as scoliosis, hyperkyphosis, spondylolisthesis or spinal stenosis. Scoliosis not only affects the spinal curvature, which can already be quantified, but also the spinal mobility and flexibility. Lonstein et al. reported that kinematic data could help to understand the nature of the scoliotic curvature as well as deciding when active treatment is necessary [25]. For postoperative follow-up, kinematic data of the spine could be also useful to determine the motion of segments above and below a spinal fusion, and may help to understand pathologies such as the adjacent segment disease. Even posture and gait changes in patients with neurological diseases such as Parkinsońs disease could be studied with rasterstereography, making it an attractive tool for further research and clinical use.

Multi-segment trunk models can be used to investigate and study trunk motion during kinematic tasks to better understand the interaction between different segments. However, for the spine a representation of motion is complex because it occurs at many different small joints, which are not all accessible for 3D motion tracking. Multiple models do exist, most of them using skin markers placed on anatomical landmarks that can then be tracked with motion-capturing systems to reconstruct the back surface [26]. Preuss and Popovic e.g. used a 6-camera VICON system to measure spine motion in seven trunk segments that were each defined by three markers placed on the back surface [27]. We adopted this triangular marker setup for our study, but in contrast to Preuss and Popovic we decreased the number of markers used in order to be able to focus on the marker detection of the two systems. The rasterstereographic method introduced in this study could be useful for kinematic trunk analysis since it uses a 3D model of the spine in addition to a back surface reconstruction. This model could potentially allow motion analysis in each segment of the vertebrae between C7 and L5. However, the goal of this present study was to evaluate the accuracy of the dynamic marker detection with rasterstereography and therefore further studies will be necessary to evaluate the spine reconstruction by a comparison with radiological methods.

The results of a previously published study by our group indicate that in dynamic conditions the use of three markers, one over the spinous process of the 7^th^ cervical vertebra and two over the lumbar dimples, are sufficient for an accurate detection of these anatomical landmarks and reconstruction of the back surface [19]. This marker setup is used because in dynamic conditions the shift of skin over the underlying bony structures can decrease the accuracy of the automatic marker detection and spine reconstruction. Rasterstereography uses the information from the back surface to draw conclusions on the position and orientation of the underlying spine. This is possible because of a correlation of the spinal symmetry line and the underlying spinous process line [28], [29]. The center of the vertebrae can then be calculated using information from a spine model created by Turner-Smith and from the orientation of the back surface above the spinous processes as well as from the information of the symmetry line [18]. Rasterstereography can also be used to calculate the vertebral rotation, as the angle between the surface orientation on the spinous process line and the normal to the frontal plane, in patients with scoliosis [30]. A further comparison of the spine reconstruction from rasterstereography with x-rays under static conditions showed a high correlation between them [28], [29]. In 1996 Farahpour stated in his work that the position of skin markers on anatomical landmarks is highly correlated with the vertebrae both in static and dynamic conditions and at all spinal levels [31]. Despite these findings, further studies are necessary to evaluate the spine reconstruction of rasterstereography and the here used spine model under dynamic conditions.

The accurate placement of markers on anatomical landmarks over bony landmarks can be difficult because of soft tissue overlaying the bony structures. Thus, the experience of the person who is placing the markers on a subject has a direct influence on the quality of the whole measurement. This influence can be reduced when the markers are placed not only by palpation but also with the help of the rasterstereographically acquired three-dimensional surface map. Therefore, we do recommend a static rasterstereographic scan for the marker placement prior to all dynamic measurements.

The results of our study indicate that an accurate detection of the placed markers under dynamic conditions is possible with rasterstereographic technique. The triangles 1–3 can be detected with ±1 mm (1–1.5%) deviation, when compared to the VICON system. Triangle 4, which extends from the spinous process of the VP down to the two lumbar dimples, has the longest sides (473 mm). For that triangle, the absolute mean difference in marker detection between rasterstereography and the VICON system was higher (±1.53 mm); however, when considering this value in relation to the length measured, the deviation is only 0.43–0.77%.

A limitation of this study is, that the accuracy of the evaluated system was done in comparison to VICON and not further evaluated with radiological studies. However, because of the dynamic setting of the measurements a validation of the here found results with x-rays is challenging. Furthermore, the radiation burden of such a study is not to be underestimated for test subjects and therefore ethically questionable. Currently, the rasterstereographic device is equipped with a single 50 frames per second digital network camera, limiting the measurements to speeds of not faster than 6 km/h (data not shown), which we used for our measurements. Upgrading the camera system to one with a higher frame rate would allow measurements with higher speeds where the subjects could run on the treadmill. Windolf et al. stated in 2008 that the performance of video capturing systems is highly dependent on camera alignment and the number of cameras used [32]. In addition marker-properties, optical projections and digital-video conversion can all potentially influence the performance of these systems [33]. It must therefore be recognized that the VICON camera alignment and flat markers used in our study could influence the precision of the VICON system. However, recognizing these potential issues, we initially evaluated the camera alignment and the flat markers in our study by comparing the VICON marker detection with a reference method (tape measurements).

In dynamic conditions, skin/soft tissue movements over the underlying bony structures are a source of error for optical measurement systems. In future work fluoroscopy or bone pins should therefore be used to investigate this problem.

## Conclusions

A novel rasterstereographic measurement device was tested that allows the determination of surface and spine topography under dynamic conditions. The accuracy of this system is comparable to the gold standard for motion detection and the measurements made under dynamic conditions can be factored into spinal models to allow a more complete analysis of the spine in various pathologies.
